# Fate of Free and Modified Forms of Mycotoxins during Food Processing

**DOI:** 10.3390/toxins12070448

**Published:** 2020-07-10

**Authors:** Michele Suman

**Affiliations:** Barilla G. R. F.lli SpA, Advanced Research Labs, via Mantova 166, 43122 Parma, Italy; michele.suman@barilla.com

International trade is highly affected by mycotoxin contaminations, which result in an annual 5% to 10% loss of global crop production [1]. In the last decade, the mycotoxin scenario has been complicated through the progressive understanding—beside emerging mycotoxins—of the parallel presence of modified (masked and conjugated) forms, in addition to the previously free known ones.

The present *Toxins* Special Issue provides original research papers and reviews that deal with the fates of all these forms of mycotoxins, with respect to aspects that cover traditional and industrial food processing, yearly grain campaign peculiar conditions and management, novel analytical solutions, consumer exposure, and biomarkers-assessment directions.

Among emerging mycotoxins, *Fusarium* ones, such as fusaproliferin (FUS), beauvericin (BEA), enniatins (ENNs), and moniliformin (MON) are discussed within the Serbian maize context by Jajić et al., highlighting exactly the economic impact of these mycotoxins in terms of the yield and quality of grain along different yearly campaigns [2]. MON, BEA and FUS are indicated as being major contaminants in more than half of the analyzed samples, and are considered in strict connection with climate change consequences which surely must be taken into account more and more in the future.

Mechanical & thermal energy involved in food processing determine changes into mycotoxin forms and the creating or destroying of new bonds with other food components: a clear example is reported by Zhang et al., describing the conversion of deoxynivalenol-3-glucoside (DON-3G) to deoxynivalenol (DON) during Chinese steamed bread processing, along the fermentation and steaming steps. Mechanical friction and shear seem to play roles which lead to these mycotoxins’ structural changes, but only in combination with other parallel factors probably related to ingredients and complex physico-chemical modifications that occur and need further investigation [3].

In a global scenario, Schaarschmidt and Fauhl-Hassek consider South American atmospheres and traditions with their review about mycotoxins’ changes during the processes of nixtamalization and tortilla production [4]. Alkaline cooking has been proven effective for reducing aflatoxins and fumonisins in cooked maize and tortillas, even if acidic conditions could partially reverse this process.

These phenomena must be deeply understood in the future for assuring that the benefits concerning the formation of low toxic hydrolyzed fumonisins are not negatively balanced out by the parallel formation of other toxicologically relevant modified and matrix-associated forms.

Remaining in the context of bakery products, can we properly design and optimize industrial baking conditions to mitigate processing contaminants and mycotoxins, while not heavily affecting the organoleptic aspects, in one single shot? The answer of Suman et al. is yes! This answer is corroborated by scientific evidence on how acrylamide concentration may be influenced by wholegrain and cocoa biscuit bakery-making parameters within a parallel strategy of DON mitigation, highlighting a significant role of pH, followed by the baking time/temperature parameters [5].

Stadler et al. focus their attention on bakery production and in particular on the optimization of recipes and processing parameters at an industrial scale, devoted to the mitigation of the main mycotoxin contaminant in the common wheat chain: DON [6]. DON degradation is accurately quantified in industrially made crackers, biscuits, and bread, showing how degradation (setting properly raising agents and baking times/temperatures) means, practically, conversion into a less toxic isomeric product (isoDON), with correspondingly positive implications towards the safety of the final consumer with regard to these commodities, with respect to the original contamination of the exploited raw material.

Moving from bakery production to meal solutions, Tittlemier et al. show changes into ergot alkaloids pattern along the durum wheat pasta production chain. More than 80% of the total ergot alkaloids are confined into outer kernel layers after milling; ergocristine, ergocristinine, and ergotamine remain the predominant components which do not also decrease after pasta production and cooking steps. Besides, the milling and cooking of pasta alters the ratio of R- to S-enantiomers; this epimerization results in a higher final concentration of the less biologically active S-enantiomers in boiled spaghetti [7].

Sueck et al. rightly consider some doubts regarding the real effect of thermal processing in food commodities such as coffee or bread; this does not always imply a degrading/detoxifying action, but, in some instances, determines the formation of unexpected forms, the toxicological definitive evaluations that would permit an adequate overall risk assessment for which are still missing. In their recent study, they specifically demonstrate the generation of the isomerization product, 20R-ochratoxin A (20R-OTA), from ochratoxin A (OTA) [8].

Ksieniewicz-Wozniak et al. are keen on the consumers’ exposure scenario, looking into the beer production chain, demonstrating that, within *Fusarium* mycotoxins, DON and its main metabolite DON-3G, among the samples analyzed, are present practically everywhere [9]. Then, nivalenol (NIV) and nivalenol-3-glucoside (NIV-3G) were also found to be largely present in both malt samples and beers. Their conclusion sounds like a warning: *Fusarium* mycotoxins should not be overlooked in countries with a very high beer consumption. In the worst-case scenario the probable daily intake (PDI) would exceed the tolerable daily intake (TDI) with only one half-liter bottle!

De Santis et al. provide relevant findings about the useful exploitation of urinary DON and its glucuronide and de-epoxydated (DOM-1) forms as biomarkers for exposure assessment purposes, permitting us to identify particularly vulnerable categories, such as children and adolescent age groups [10].

Finally, there is the evident necessity for all the stakeholders (from authorities, to control bodies and food business operators) to dispose of rapid and easy-to-use methods for the determination of free and modified forms of toxins in raw materials; Lippolis et al. propose here a fluorescence polarization immunoassay (FPIA) for the simultaneous determination of T-2 toxin, HT-2 toxin and relevant glucosides, expressed as sum, exploiting a HT-2-specific antibody with high sensitivity and high cross-reactivity towards the different forms present in cereals [11]. This analytical method is compliant with harmonized guidelines for the validation of screening methods recently stated by European regulations.

I would like to congratulate and thank all the authors involved in this special issue of *Toxins* and I hope you enjoy reading its contents; it gives a taste of an exciting scientific field which has several implications into our daily life, because (i) it covers our diet practically and from every point of view, (ii) it intersects our culinary uses and customs, but also industrial production processes, and (iii) it involves a careful evaluation of costs and benefits and an constant continuous improvement of mitigation strategies. There will still be a lot to see and discover in the coming years!

## References

[1] EC (2015). European Commission Decision C 2453: Horizon 2020 Work Programme 2014–2015: Food Security, Sustainable Agriculture and Forestry, Marine and Maritime and Inland Water Research and Bioeconomy (Revised). https://ec.europa.eu/research/participants/data/ref/h2020/wp/2014_2015/main/h2020-wp1415-food_en.pdf.

[2] Jajić I., Dudaš T., Krstović S., Krska R., Sulyok M., Bagi F., Savić Z., Guljaš D., Stankov A. (2019). Emerging Fusarium Mycotoxins Fusaproliferin, Beauvericin, Enniatins, and Moniliformin in Serbian Maize. Toxins.

[3] Zhang H., Wu L., Li W., Zhang Y., Li J., Hu X., Sun L., Du W., Wang B. (2020). Conversion of Deoxynivalenol-3-Glucoside to Deoxynivalenol during Chinese Steamed Bread Processing. Toxins.

[4] Schaarschmidt S., Fauhl-Hassek C. (2019). Mycotoxins during the Processes of Nixtamalization and Tortilla Production. Toxins.

[5] Suman M., Generotti S., Cirlini M., Dall’Asta C. (2019). Acrylamide Reduction Strategy in Combination with Deoxynivalenol Mitigation in Industrial Biscuits Production. Toxins.

[6] Stadler D., Lambertini F., Woelflingseder L., Schwartz-Zimmermann H., Marko D., Suman M., Berthiller F., Krska R. (2019). The Influence of Processing Parameters on the Mitigation of Deoxynivalenol during Industrial Baking. Toxins.

[7] Tittlemier S.A., Drul D., Roscoe M., Turnock D., Taylor D., Fu B.X. (2019). Fate of Ergot Alkaloids during Laboratory Scale Durum Processing and Pasta Production. Toxins.

[8] Sueck F., Hemp V., Specht J., Torres O., Cramer B., Humpf H.-U. (2019). Occurrence of the Ochratoxin A Degradation Product 2′R-Ochratoxin A in Coffee and Other Food: An Update. Toxins.

[9] Ksieniewicz-Woźniak E., Bryła M., Waśkiewicz A., Yoshinari T., Szymczyk K. (2019). Selected Trichothecenes in Barley Malt and Beer from Poland and an Assessment of Dietary Risks Associated with their Consumption. Toxins.

[10] De Santis B., Debegnach F., Miano B., Moretti G., Sonego E., Chiaretti A., Buonsenso D., Brera C. (2019). Determination of Deoxynivalenol Biomarkers in Italian Urine Samples. Toxins.

[11] Lippolis V., Porricelli A.C.R., Mancini E., Ciasca B., Lattanzio V.M.T., De Girolamo A., Maragos C.M., McCormick S., Li P., Logrieco A.F. (2019). Fluorescence Polarization Immunoassay for the Determination of T-2 and HT-2 Toxins and Their Glucosides in Wheat. Toxins.

